# Retraction: Targeting mitochondria to protect the heart: a matter of balance?

**DOI:** 10.1042/CS20200236_RET

**Published:** 2025-08-13

This commentary is being retracted from *Clinical Science* at the request of the authors, the Editor-in-Chief and the Editorial Board, and a new article is being submitted. This follows the retraction of the article “Balancing mitochondrial dynamics via increasing mitochondrial fusion attenuates infarct size and left ventricular dysfunction in rats with cardiac ischemia/reperfusion injury” Maneechote et al., Clin Sci (Lond) (2019) 133 (3): 497–513 (DOI:10.1042/cs20190014), upon which this commentary was based.

The authors of this commentary had no involvement in the retracted research article by Maneechote et al., and the retraction of this associated commentary is based solely on the Journal having lost confidence in the validity of the scientific data presented in the original research paper. The authors agree with the Journal’s decision.

